# Bone Marrow Mesenchymal Stem Cells Stimulate Proliferation and Neuronal Differentiation of Retinal Progenitor Cells

**DOI:** 10.1371/journal.pone.0076157

**Published:** 2013-09-30

**Authors:** Jing Xia, Min Luo, Ni Ni, Junzhao Chen, Yamin Hu, Yuan Deng, Jing Ji, Jibo Zhou, Xianqun Fan, Ping Gu

**Affiliations:** Department of Ophthalmology, Shanghai Ninth People’s Hospital, School of Medicine, Shanghai Jiao Tong University, Shanghai, China; Rutgers - New Jersey Medical School, United States of America

## Abstract

During retina development, retinal progenitor cell (RPC) proliferation and differentiation are regulated by complex inter- and intracellular interactions. Bone marrow mesenchymal stem cells (BMSCs) are reported to express a variety of cytokines and neurotrophic factors, which have powerful trophic and protective functions for neural tissue-derived cells. Here, we show that the expanded RPC cultures treated with BMSC-derived conditioned medium (CM) which was substantially enriched for bFGF and CNTF, expressed clearly increased levels of nuclear receptor TLX, an essential regulator of neural stem cell (NSC) self-renewal, as well as betacellulin (BTC), an EGF-like protein described as supporting NSC expansion. The BMSC CM- or bFGF-treated RPCs also displayed an obviously enhanced proliferation capability, while BMSC CM-derived bFGF knocked down by anti-bFGF, the effect of BMSC CM on enhancing RPC proliferation was partly reversed. Under differentiation conditions, treatment with BMSC CM or CNTF markedly favoured RPC differentiation towards retinal neurons, including Brn3a-positive retinal ganglion cells (RGCs) and rhodopsin-positive photoreceptors, and clearly diminished retinal glial cell differentiation. These findings demonstrate that BMSCs supported RPC proliferation and neuronal differentiation which may be partly mediated by BMSC CM-derived bFGF and CNTF, reveal potential limitations of RPC culture systems, and suggest a means for optimizing RPC cell fate determination in vitro.

## Introduction

Visual impairment, including retinitis pigmentosa, age-related macular degeneration, glaucoma and diabetic retinopathy, severely affects the quality of life of patients and their families. These retinal disorders are all characterised by the dysfunction and loss of retinal neurons, leading to an irreversible decline in visual function, and there are at present no effective restorative therapies available for these diseases [1,2]. Cell replacement therapy is a promising therapeutic approach to restoring visual function to the abnormal retina and has become an important strategy in retinal regeneration research.

Retinal progenitor cells (RPCs) are a subset of undifferentiated cells that have the ability to self-renew and the potential to differentiate into various retinal neurons [3]. They are capable of cytoarchitectural integration and differentiation towards cells expressing characteristic markers of retinal neurons, thereby improving visual function in the host [3,4]. These findings suggest that RPCs may be able to replace degenerating retinal cells. Although these studies described promising therapeutic applications of RPCs, there are numerous related issues and concerns, including the improvement of proliferation capacity and the preferential differentiation into specific neurons but not glial cells. These concerns must be addressed for the successful use of RPCs in cell replacement therapy in the future. One way to explore the promise of RPCs is to modify culture conditions in an attempt to improve the potential of RPC proliferation and differentiation. Among the most accessible strategies is an attractive strategy of co-culture that has been considered to improve the proliferation and differentiation of progenitor cells [5-7].

Bone marrow mesenchymal stem cells (BMSCs) have attracted much attention because they can be readily obtained through a well-established procedure and are relatively simple to isolate and expand *in vitro*. A previous series of reports indicated that BMSCs have been shown to provide a powerful neuroprotection in degenerative disorders of the central nervous system. BMSCs were able to secrete neurotrophic factors and anti-inflammatory cytokines, which are potentially therapeutic in models of Huntington's disease, Parkinson’s disease, glaucoma and light-damaged retina [8-10]. In addition, previous studies also indicated that BMSCs can provide instructive signals to direct the differentiation of neural stem cells (NSC) and promote axonal development when BMSCs and NSCs are co-cultured *in vitro* [11].

Here, we investigated the communication between BMSC-derived CM and RPCs, and demonstrate that compared to untreated RPCs, BMSC CM-treated RPCs displayed clearly enhanced expression of nuclear receptor TLX (an essential regulator of NSC self-renewal) and betacellulin (BTC, an EGF-like protein reported to support NSC proliferation and enhance neurogenesis), and exhibited a large capacity to stimulate RPC proliferation and enhance RPC neuronal differentiation *in vitro*.

## Experimental Procedures

### Experimental animals

All animal procedures used in the present study were performed according to the ARVO statement for the Use of Animals in Ophthalmic and Vision Research and were approved by the Ethics Committee of Shanghai Ninth People’s Hospital affiliated with the Shanghai Jiao tong University School of Medicine.

### Isolation and culture of retinal progenitor cells

RPCs were isolated from the neural retina of postnatal day 1 GFP transgenic C57BL/6 mice [12,13]. Briefly, retinas were harvested and subjected to several cycles of 0.1% type I collagenase (Invitrogen, Carlsbad, CA) digestion. The cell suspensions were then forced through a nylon mesh with 100-µm pores, centrifuged, and resuspended in standard culture medium (SM), containing advanced DMEM/F12 (Invitrogen), 1% N2 neural supplement (Invitrogen), 2 mM L-glutamine (Invitrogen), 100 U/ml penicillin-streptomycin (Invitrogen), and 20 ng/ml epidermal growth factor (EGF, Invitrogen). The cells were seeded into flasks at a density of 2×10^5^ cells/ml with SM. Spherical clusters appeared within the first 2 or 3 days. The culture media were changed every 2 days, and proliferating cells were passaged at regular intervals of 3-4 days.

### Bone marrow mesenchymal stem cells (BMSCs) culture

BMSCs were isolated from the femur and tibia of 4-week-old male Sprague Dawley rats (Shanghai Experimental Animal Center), as described [14]. Briefly, the ends of the bones were cut, and the marrow was extruded with α-MEM medium using a needle and syringe. The cell suspensions were then centrifuged, all of the marrow cells were plated on 25-cm^2^ plastic flasks in α-MEM medium, supplemented with 10% foetal bovine serum (FBS, Invitrogen), 100 U/ml penicillin and 100 mg/ml streptomycin. These cells were then incubated at 37 °C with 5% CO_2_. After seeding for 12 h, the non-adherent cells were removed by replacing the medium. The medium was added and replaced every 3 or 4 days. When the cells grew to confluence, they were harvested with 0.25% trypsin and were replated on 25-cm^2^ plastic flasks, again cultured to confluence and harvested. All of the experiments described below were performed using cells from the third to the fifth passage.

### Neural stem cell (NSC) culture

Mouse NSCs were kindly provided by Dr T. Q. Wen and were cultured in DMEM with 10% FBS, 5% horse serum (Invitrogen), 1 mM glutamine, 100 U/mL penicillin and 100 mg/mL streptomycin, as previously described [15].

### Lens epithelial cell culture

Lens epithelial cells were isolated from the eye of the mature rabbit [16]. The anterior lens capsule was torn with tweezers and scissors and was incubated in DMEM/F12 supplemented with 10% FBS, 100 U/ml penicillin, and 100 mg/ml streptomycin (complete medium) at 37 °C with 5% humidified CO_2_ for 24 h. Then, the capsules were digested with 0.25% trypsin for 10 min, centrifuged, and resuspended in complete medium. After seeding for 48 h, the non-adherent cells were removed by replacing the medium. The medium was added and replaced every 3 or 4 days. When the cells grew to confluence, they were harvested with 0.25% trypsin and were replated on plastic flasks.

### Preparation of conditioned media and ELISA analysis

When the BMSCs, lens epithelial cells, RPCs and NSCs were grown to 70–80% confluence in 25-cm^2^ plastic flasks, the culture media were replaced with SM (but not including 20 ng/ml EGF). After 12 h, the conditioned media were collected and sterile-filtered through a 0.2-μm membrane and conditioned samples were stored at -80 °C before use. For ELISA analysis, ELISA was performed using a Mouse bFGF ELISA kit (abcam INC., Cambridge, MA) and a Rat CNTF ELISA kit (abcam INC.), and their protocols. As a reference for quantification, a standard curve was established by a serial dilution for bFGF protein (18.8 pg/ml-2.0 ng/ml) and CNTF protein (8.23pg/ml-2.0 ng/ml).

### Proliferation and differentiation studies of RPCs

For the proliferation experiments, the RPCs were seeded in flasks at a density of 2×10^5^ cells/ml under different conditions: CM from BMSCs, lens or NSCs (all supplemented with 20 ng/ml EGF), or the SM with or without 1.2 ng/ml bFGF (Invitrogen) or 1.2 ng/ml CNTF (Chemicon, Temecula, CA), or BMSC CM plus 2.5 ug/ml anti-bFGF (Millipore, Temecula, CA) or 2.5 ug/ml anti-CNTF (R & D Systems, Minneapolis, MN). For the differentiation experiments, the RPCs were cultured with the standard differentiation medium (SDM) consisting of advanced DMEM/F12, 1% N2 neural supplement, 2 mM L-glutamine, 100 U/ml penicillin-streptomycin and 5% FBS, or SDM with 1.2 ng/ml bFGF or 1.2 ng/ml CNTF, or the CM from BMSCs, lens or NSCs supplemented with 5% FBS, or BMSC CM plus 2.5 ug/ml anti-bFGF or 2.5 ug/ml anti-CNTF containing 5% FBS. For all of the culture conditions, the media were half changed daily.

### Cell viability

The effect of CM from the BMSCs, NSCs and lens epithelial cells on RPC proliferation was assessed using the cell counting kit-8 (CCK-8) (Dojindo, Kumamoto, Japan) [17]. Briefly, RPCs were suspended at a final concentration of 1×10^4^ cells/well and cultured in 96-well plates. At days 1, 4 and 7 of the culture period, the CCK-8 solution was added to each well. After cells were incubated for another 3 h at 37 °C according to the reagent instructions, the absorbance at 490 nm was measured using an ELISA microplate reader (ELX800, BioTeK, USA). Cell viability is in direct proportion to the absorbance at 490 nm; therefore, viability was expressed as the A490 value.

### Reverse transcription and quantitative polymerase chain reaction (qPCR)

Total RNA was extracted from each sample using the RNeasy Mini Kit (Qiagen, Valencia, CA) and synthesised first-strand complementary cDNA using the PrimeScript™ RT reagent kit (Perfect Real Time, TaKaRa, Dalian, China) [13]. The resulting cDNAs were diluted 20-fold in nuclease-free water (Invitrogen) and used as templates for qPCR. qPCR was carried out in a 20-µl solution containing 10 µl reaction mixture, 2 µl of cDNA, and 300 nM of gene-specific primers (Table 1) designed using Primer 3 software. The qPCR was conducted using a 7500 Real-Time PCR Detection System (Applied Biosystems, Irvine, CA) and activated at 95°C for 10 min and 40 cycles of amplification (15 s at 95°C and 1 min at 60°C). The efficiency of the reaction was measured with primers using serial dilutions of the cDNA (1:1, 1:5, 1:25, 1:125, 1:625 and 1:3,125). Each sample was tested in triplicate. The relative gene expression levels were analysed using the Pfaffl method [18]. The data are expressed as fold change relative to untreated controls after normalizing to β-actin.

**Table 1:** Primers used for quantitative RT-PCR.

Genes	Accession no.	Forward (5’–3’)	Reverse (5’–3’)	Annealing temperature (°C)	Product size (base pairs)
Pax6	NM_013627	agtgaatgggcggagttatg	acttggacgggaactgacac	60	132
Mash1	NM_008553	tctcctgggaatggactttg	ggttggctgtctggtttgtt	60	142
Nestin	NM_016701	aactggcacctcaagatgt	tcaagggtattaggcaagggg	60	235
Vimentin	NM_011701	tggttgacacccactcaaaa	gcttttggggtgtcagttgt	60	269
Ki-67	X82786	cagtactcggaatgcagcaa	cagtcttcaggggctctgtc	60	170
Betacellulin	NM_007568	cacaggtaccacccctagaca	cctttctcacagatgcaggag	60	135
TLX	NM_152229.2	cgattagacgccactgaa	ggtatctggtatgaatgtagc	60	166
Map2	NM_001039934	agaaaatggaagaaggaatgactg	acatggatcatctggtaccttttt	60	112
*β3*-tubulin	NM_023279	cgagacctactgcatcgaca	cattgagctgaccagggaat	60	152
PKC-*α*	NM_011101	cccattccagaaggagatga	ttcctgtcagcaagcatcac	60	212
AP2*α*	NM_001122948.1	gccgtccacctagccaggga	gattgggccgcgagttcccc	60	208
Brn3a	NM_011143.4	cgctctcgcacaacaacatga	ttcttctcgccgccgttga	60	121
GFAP	NM_010277	agaaaaccgcatcaccattc	tcacatcaccacgtccttgt	60	184
Rhodopsin	NM_145383	tcaccaccaccctctacaca	tgatccaggtgaagaccaca	60	216
β-actin	NM_007393	agccatgtacgtagccatcc	ctctcagctgtggtggtgaa	60	152

### Immunohistochemistry

The proliferation and differentiation of RPCs were studied under the four culture conditions described above. RPCs were cultured on glass coverslips (VWR, West Chester, PA) coated with laminin (Sigma-Aldrich, Saint Louis, MO) and were fixed in 4% (w/v) paraformaldehyde (Sigma-Aldrich) in 0.1 M phosphate-buffered saline (PBS; 2.68 mM KCI, 1.47 mM KH2PO4, 135.60 mM NaCl and 8.10 mM Na2HPO4) for 15 min at room temperature. The cells were then washed with PBS and blocked for 1 h in a blocking solution (PBS containing 10% (v/v) normal goat serum (Invitrogen)), 0.3% Triton X-100 (Sigma-Aldrich) and 0.1% NaN3 (Sigma-Aldrich). The samples were then incubated in the primary antibodies (Table 2) at 4 °C overnight. All of the identiﬁed protein markers and their properties are classiﬁed and listed in Table 2. After washing with PBS solution and incubated in ﬂuorescent secondary antibodies (Alexa Fluor546 goat anti-mouse or goat anti-rabbit, 1:800 in PBS, BD) for 1 h at room temperature. The cell nuclei were counterstained with 4', 6-diamidino-2-phenylindole (DAPI) (Vector Laboratories, Burlingame, CA). Immunopositive cells were detected using a ﬂuorescent microscope (Olympus BX51, Japan). The control samples were processed using the same protocol but with the omission of the primary antibody.

**Table 2:** Primary antibodies used for immunocytochemistry.

Antibodies	Type	Specificity in retina	Source	Dilution
Nestin	Mouse monoclonal	Progenitors reactive glia	BD	1:200
Ki-67	Mouse monoclonal	Proliferating cells	BD	1:200
Map2	Rabbit monoclonal,	Neurons	Epitomics	1:200
*β*3-tubulin	Mouse monoclonal	Neurons	Chemicon	1:100
AP2*α*	Mouse monoclonal	Amacrine cells	DSHB	1:600
Brn3a	Rabbit polyclonal	Ganglion cells	Millipore	1:500
PKC-α	Mouse monoclonal	Bipolar cells	BD	1:200
GFAP	Mouse monoclonal	Glia	Chemicon	1:200
Rhodopsin	Mouse monoclonal	Photoreceptors (rods)	Chemicon	1:100

### Cell counts

The cell counts were performed using a ﬂuorescent microscope (Olympus BX51, Japan) and Image pro plus 6.0 (Media Cybernetics, Roper, GA). The quantification of cells was based on counting the number of DAPI-stained nuclei and immunoreactive cells in at least 5 independent fields, for a total of at least five hundred to one thousand cells.

### Statistical analysis

The results represent the average of three experiments, and the data are presented as the mean ± SD. Each experiment was performed at least three times unless otherwise specified. Statistical significance was determined using unpaired Student’s t-test, and a value of *P<0.05 was considered to be statistically significant.

## Results

### Morphology and expansion potential of CM-treated RPCs

**Figure 1:**
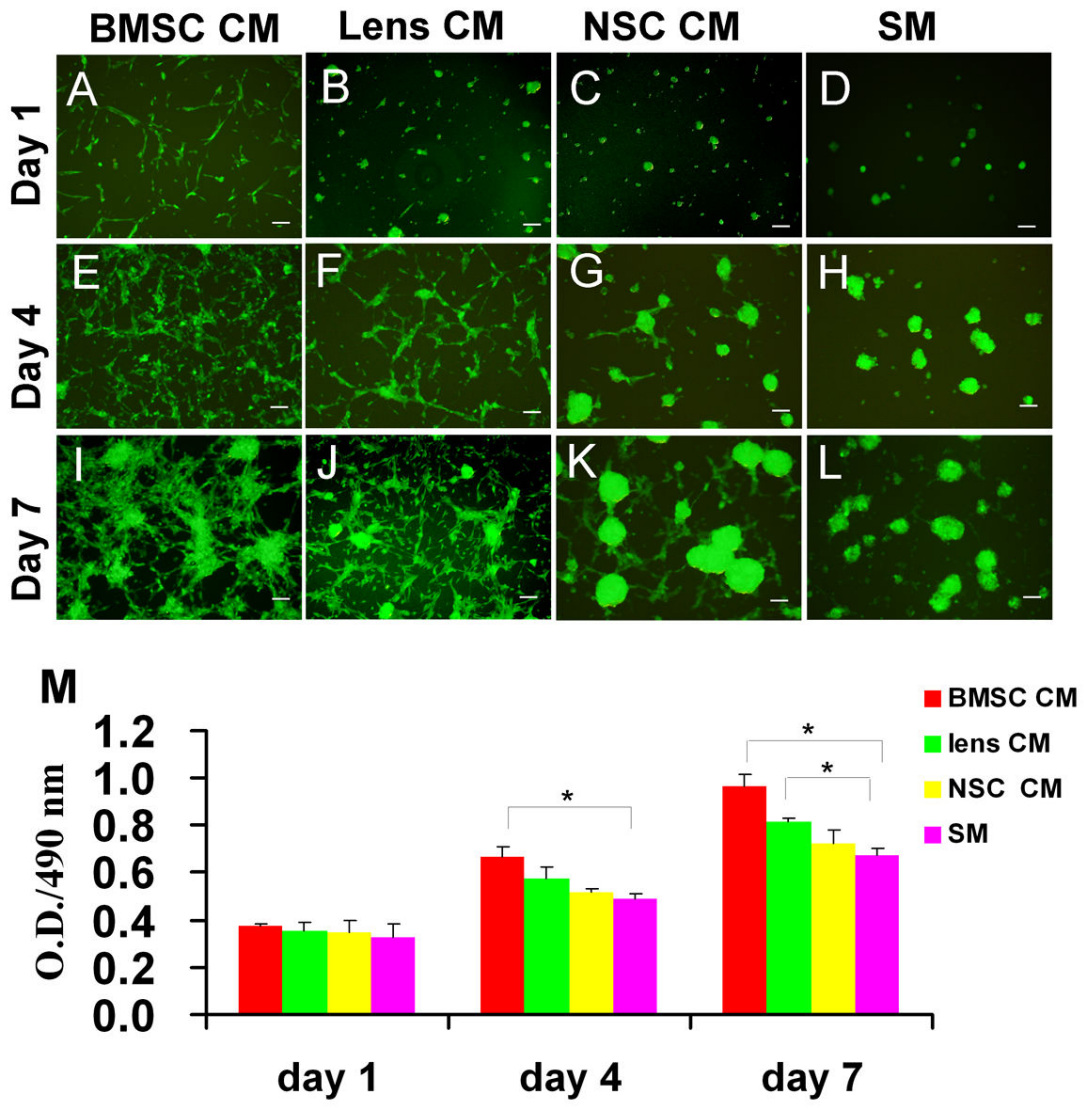
Morphology and proliferation capacity of CM-treated RPCs. In the proliferation conditions, the RPCs were cultured in BMSC, lens and NSC CM, and morphology images were taken on days 1 (A, B, C, D), 4 (E, F, G, H) and 7 (I, J, K, L). In the presence of BMSC CM, the cells attached to the surface of the flask and extended short processes by the first day; with time, most cells exhibited two or more long processes and formed an intercellular network by day 7 (A, E, I). With lens CM, the cells were adherent, and short processes extending from a few cells were observed by day 1; with time, most cells extended processes, but there were fewer adherent cells than in the BMSC CM condition (B, F, J). In the presence of NSC CM (C, G, K) or SM, (D, H, L), most RPCs grew as spherical clusters which adhered to the flask or floated in the culture medium. The spherical cluster size in NSC CM was larger than that in SM. The expansion potential of the RPC cultures was assessed using CCK-8 analysis. The cells exhibited an obvious increase in expansion potential in CM cultures, especially in the BMSC CM, than in SM cultures (M). Scale bars: 100 µm.

Under proliferation conditions, the RPCs were cultured in BMSC, lens and NSC CM, and morphology images were taken on days 1, 4 and 7 (Figure 1). In the presence of BMSC or Lens CM, the RPCs grew as an adherent monolayer of cells or cellular clusters. In BMSC CM, most of the cells extended short processes by the first day; with time, most cells exhibited two or more long processes that formed a network between cells by day 7 (Figure 1A, E, I). Under the Lens CM, short processes extended from a few cells were observed by day 1, more cells extended processes with time, and their were fewer adherent cells compared with the cultures in BMSC CM (Figure 1B, F, J). In response to NSC CM (Figure 1C, G, K) or SM (Figure 1D, H, L), most RPCs grew as spherical clusters that adhered to the uncoated flask or floated in the culture medium. The spherical clusters grew with time, but the average spherical cluster size in NSC CM was larger than that observed in SM.

To investigate the effect of CM on RPC proliferation, the proliferation capacity of the cells exposed to CM was evaluated by cell counting kit-8 (CCK-8) analysis. In comparison with the control, RPCs cultured in the presence of CM, especially BMSC CM, exhibited obvious increases in expansion capacity (Figure 1M).

In addition, our ELISA analysis exhibited that the BMSC CM was substantially enriched for bFGF and CNTF compared to RPC-cultured SM (Figure S1). When cultured in the presence of bFGF in SM, RPCs displayed enhanced proliferation capacity (Figure S2A), while the obvious effect of the addition of CNTF to SM on RPC expansion was not detected (Figure S2B). After BMSC CM-derived bFGF knocked down by anti-bFGF, the effect of BMSC CM on enhancing RPC proliferation was partly reversed (Figure S2). These results suggest that the effect of BMSC CM on RPC expansion may be partly mediated by BMSC CM–derived bFGF.

### Quantitative evaluation of the effect of CM on progenitor and proliferation marker expression in proliferating RPCs

**Figure 2:**
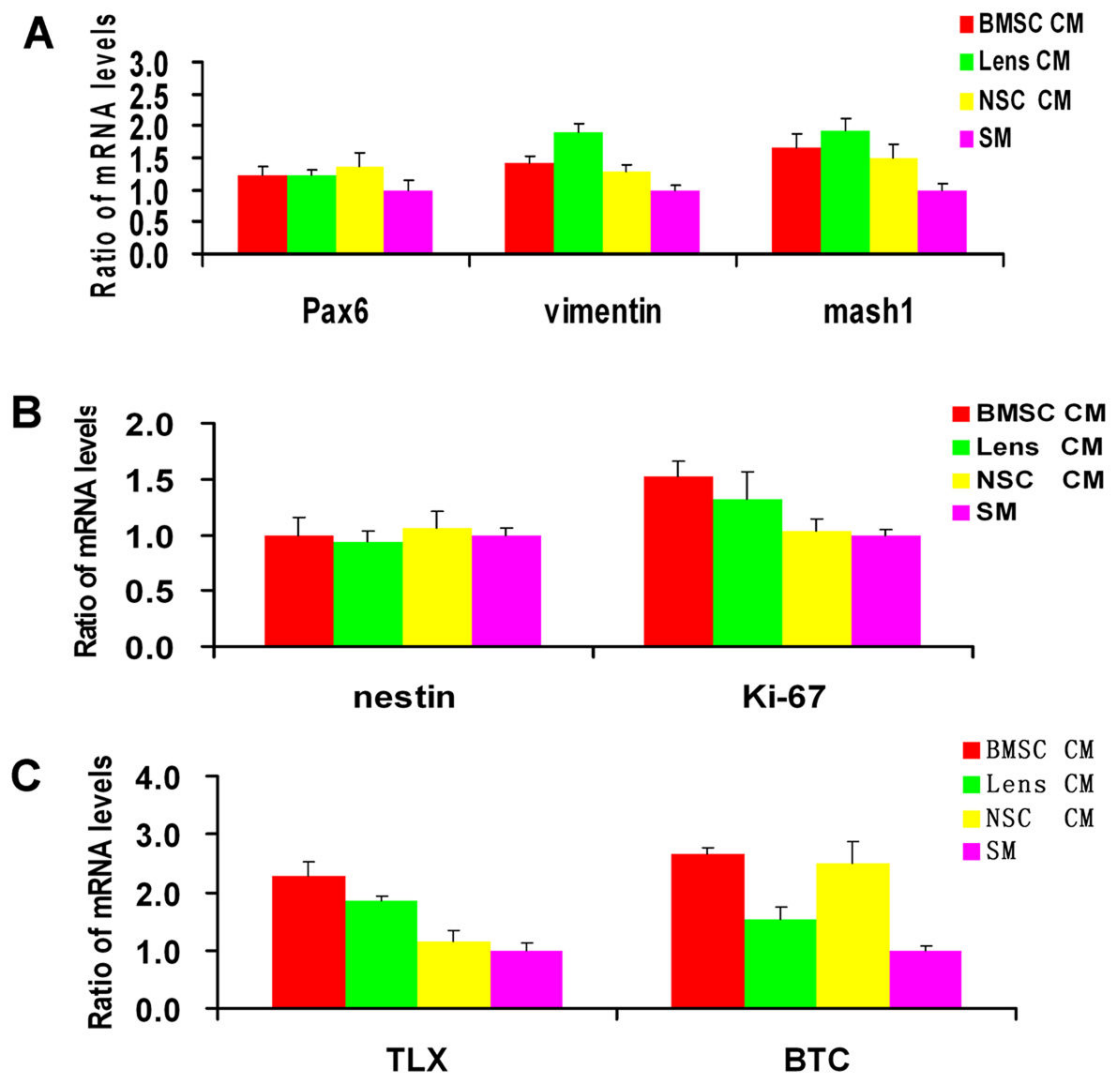
qPCR analysis of progenitor and proliferation marker expression in RPCs under proliferation conditions. The qPCR results showed that the expression levels of retinal progenitor markers, including Pax6, vimentin and Mash1 (A) were significantly or marginally higher in the RPCs treated with CM than in those cultured with SM. No obvious changes were detected in the expression of nestin (a retinal progenitor marker) between different groups; however, the expression levels of ki-67 (a cell proliferation marker) were slightly higher in the three CM-treated cultures (B). In addition, RPCs showed significantly higher expression levels of TLX (a nuclear receptor) and BTC (an EGF-like protein) in response to BMSC CM treatment than in the other groups (C).

qPCR was used to determine the expression of critical retinal progenitor-related markers, including nestin, vimentin, PAX6 and Mash1, which play important roles in retinal development, in CM-treated RPCs. The qPCR results showed that the levels of vimentin, PAX6 and Mash1 were significantly or marginally higher in CM-treated RPC cultures than in SM cultures (Figure 2A). No obvious change was detected in the expression of nestin between different groups. Meanwhile, the expression levels of the cell proliferation marker ki-67 were slightly upregulated in the CM-treated cells, especially in the BMSC CM-treated RPC cultures (Figure 2B). In response to CM treatment, especially with BMSC-CM, we also found high expression levels of TLX and BTC, which have reported to play important roles in neural stem cell proliferation (Figure 2C). These results indicated that the proliferation of RPCs can be enhanced by BMSC CM treatment.

### The expression levels of progenitor- and proliferation-related markers by immunocytochemistry analysis

**Figure 3:**
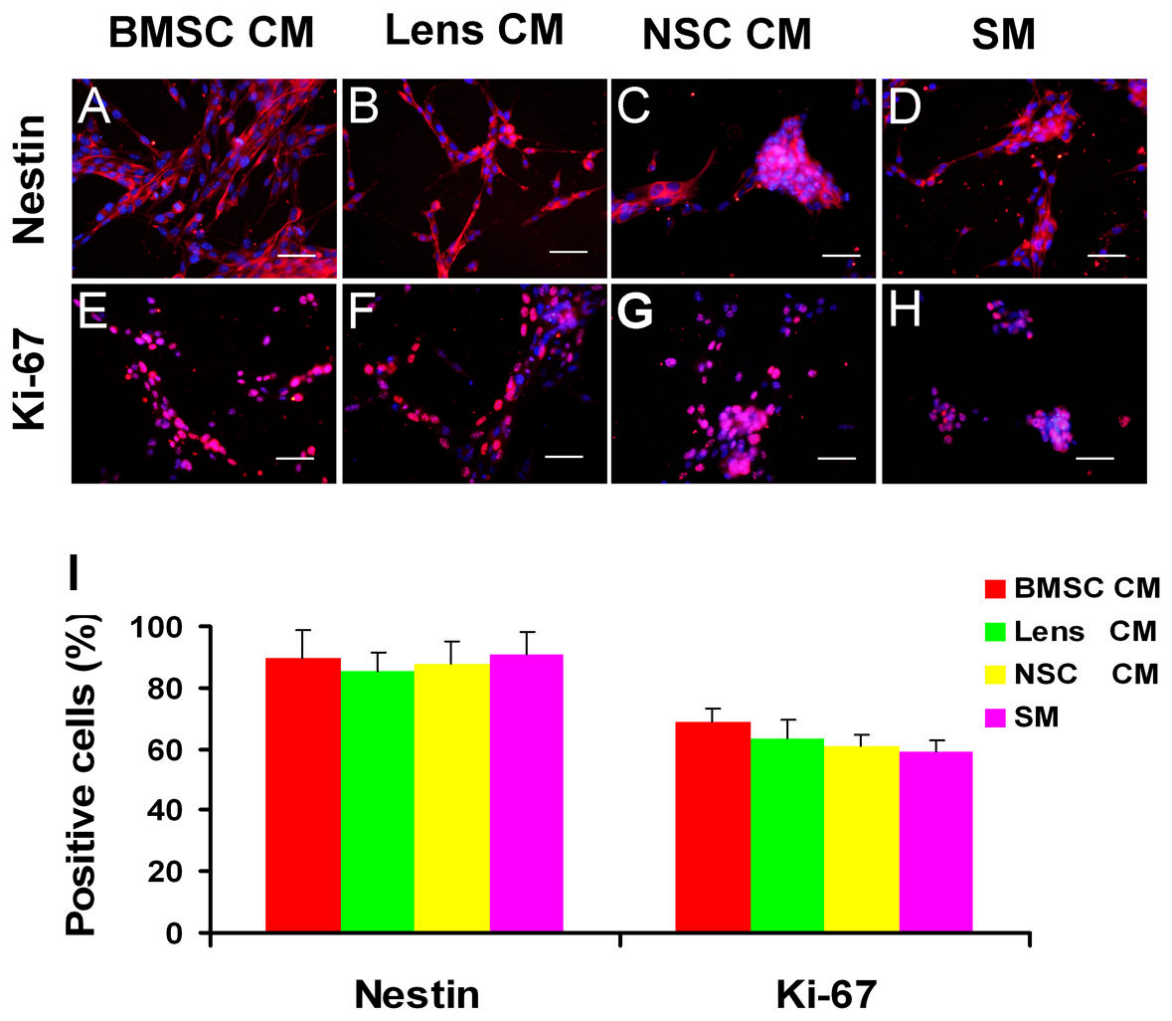
Immunostaining analysis of the expression of progenitor- and proliferation-related markers. After four days of culture in proliferation conditions, the cells were fixed and immunostained with antibodies against nestin (A-D) and ki-67 (E-H). The ratio of the nestin-positive is similar between different groups, while the percentages of ki-67-positive cells were high in the RPC cultures in BMSC CM, Lens CM and NSC CM, when compared with the cultures in SM condition (I). The percentage of positive cells was determined by dividing the number of immunopositive cells by the number of nuclei stained with DAPI. Five hundred to one thousand cells for each RPC subgroup and each culture were counted in random fields. Scale bars: 50 µm.

Immunocytochemistry was performed to evaluate the expression of progenitor- and proliferation-associated markers (Figure 3). Our data showed that there were no significant differences in the proportions of nestin-positive cells between different groups, while the percentages of ki-67-positive cells were high in the RPC cultures treated with CM, especially those treated with BMSC CM (69.1 ± 4.1%), when compared with the controls (58.9 ± 3.5%) (Figure 3A-I). Even in RPCs treated with CM for up to 3 passages, the percentages of nestin- and Ki-67-positive cells were sustained. In addition, cells that were immunoreactive for the differentiated cell markers β3-tubulin, MAP-2, protein kinase C alpha (PKC-α), rhodopsin and glial fibrillary acidic protein (GFAP) were not detected in either the CM-treated RPC cultures or the control cells (data not shown). These data suggest that the undifferentiated state of RPCs was unaffected by exposure to BMSC CM and that the BMSC CM can stimulate RPC proliferation.

### Effect of CM on RPC morphology and gene expression under differentiation conditions

**Figure 4:**
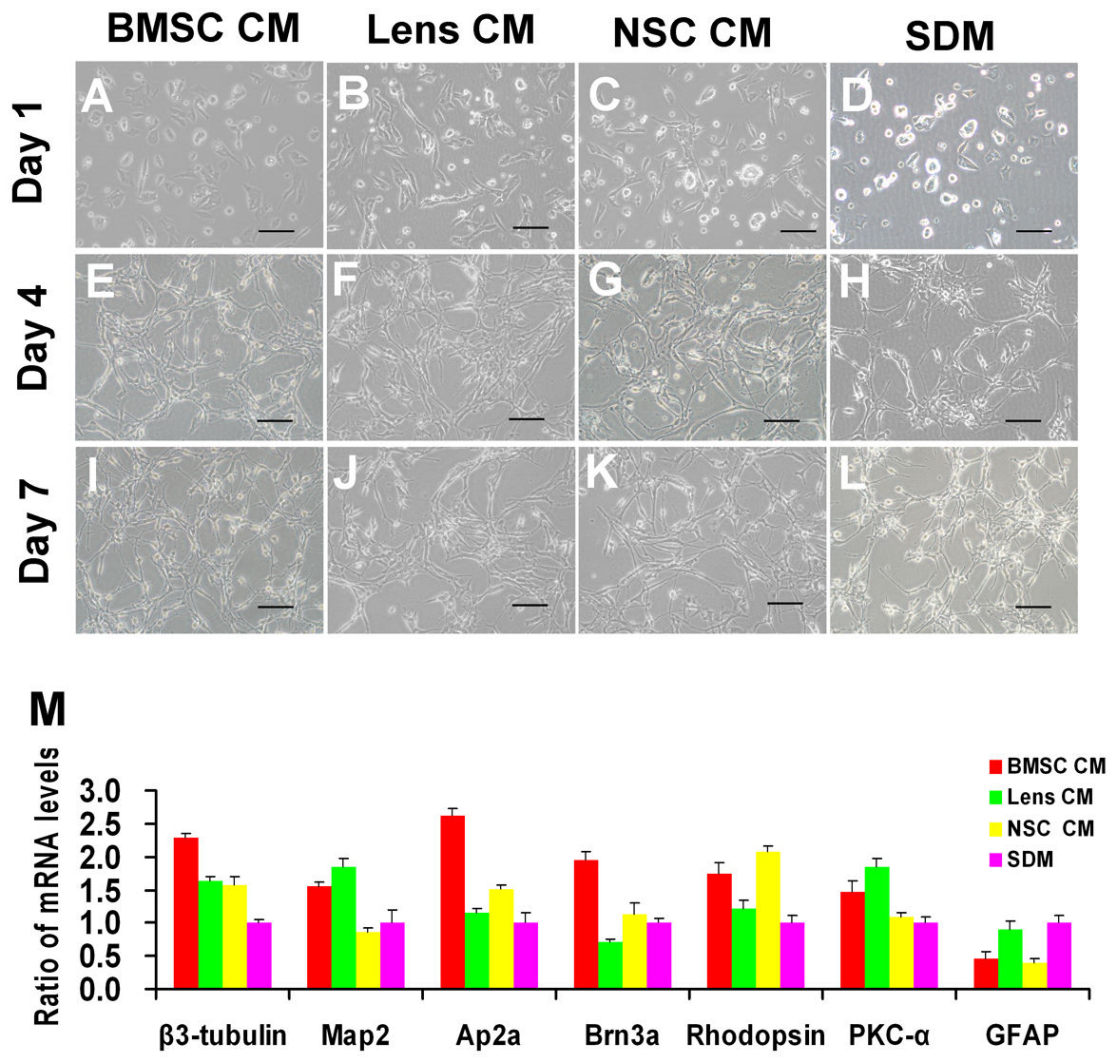
Morphology and gene expression levels of RPCs under differentiation conditions. One day after the cells were cultured in the differentiation conditions, the cells in the differentiation medium without CM only occasionally extended short processes (D), whereas most of the cells extended short processes in the CM-treated cultures (A, B, C). Under differentiation conditions, RPCs treated with BMSC CM (A, E, I), lens CM (B, F, J), NSC CM (C, G, K) and SDM (D, H, L) typically exhibited increasing neurite-like cellular processes and formed a network among the cells with time. However, the cellular processes of RPC cultures treated with CM, especially with BMSC CM, were longer and appeared more numerous than in the control cultures (without CM treatment). In the qPCR analysis (M), a notable up-regulation in the expression of β3-tubulin, activator protein 2 alpha (Ap2α, an amacrine cell marker) and Brn3a (a ganglion cell marker) was detected in the BMSC CM-treated RPC cultures. The levels of the neuronal markers MAP2 and PKC-α (a marker for bipolar cells) were significantly higher in the BMSC CM- and lens CM-treated RPC cultures compared with the control. The expression levels of rhodopsin (a photoreceptor marker) were higher in RPC cultures treated with BMSC CM or NSC CM. In addition, low expression levels of the glial marker GFAP were found in the RPC cultures treated with CM. Scale bars: 100 µm.

In the differentiation conditions, the RPCs in all of the groups differentiated into cells with divergent morphologies and neurite-like processes (Figure 4A-L). The cells in differentiation medium without CM only occasionally extended short processes, while most of the RPCs extended short processes in CM-treated cultures within the first day of culture in the differentiation conditions (Figure 4A-D). The cultured cells typically exhibited increasing neurite-like cellular processes that formed an intercellular network over time; however, the cellular processes of RPC cultures treated with CM, especially BMSC CM, appeared longer and more numerous than those of RPCs cultured without CM (Figure 4E-L).

qPCR analysis was carried out to investigate the fate potential of RPCs exposed to CM. A notable upregulation in the expression of β3-tubulin, activator protein 2 alpha (Ap2α, an amacrine cell marker) and Brn3a (a ganglion cell marker) was detected in the BMSC CM-treated RPC cultures (Figure 4M). Neuronal markers MAP-2 and PKC-α (a marker for bipolar cells) were significantly higher in BMSC CM- and Lens CM- treated RPC cultures compared with the control. The expression levels of rhodopsin (a photoreceptor cell marker), which was of the most interest to us, were significantly raised in the BMSC and NSC CM-treated RPC cultures (Figure 4-M). In addition, low expression levels of the glial cell marker GFAP were displayed in the RPC cultures in response to BMSC CM treatment (Figure 4M). These results suggest that the BMSC CM-treated RPCs have a greater potential for generating retinal neurons.

In addition, when BMSC CM-derived CNTF knocked down by anti-CNTF, the effect of BMSC CM on enhancing RPC differentiation towards above retinal neurons were slightly inhibited; While the addition of CNTF to the RPC cultures in SDM, retinal neuronal marker expression levels of MAP-2, AP2α, Brn3a and rhodopsin were obviously upregulated (Figure S3). The effect of BMSC CM-derived bFGF on RPC differentiation was not detected (Data not shown), indicating the effect of BMSC CM on RPC differentiation may be at least partly mediated by BMSC CM-derived CNTF.

### Multipotentiality of CM-treated RPCs

**Figure 5:**
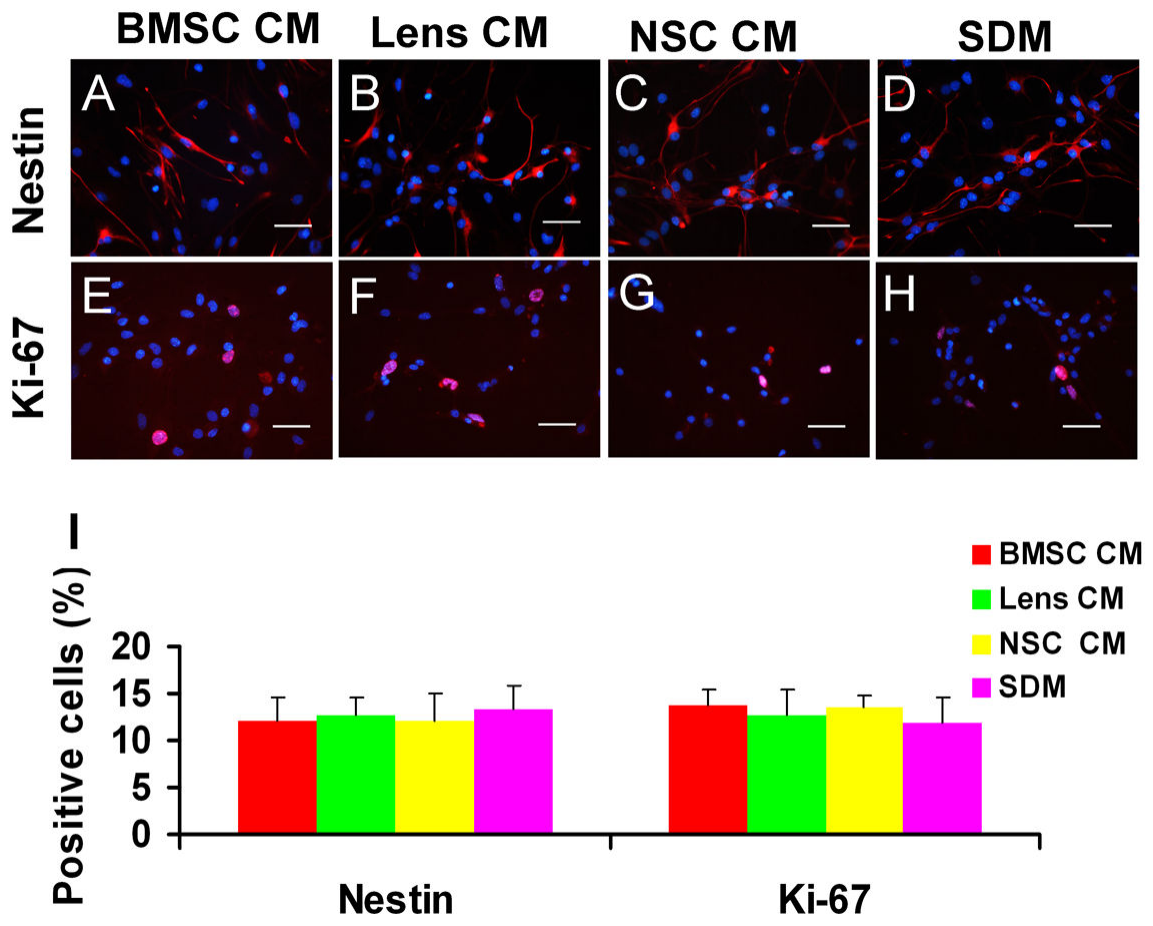
Progenitor and proliferation marker expression of RPC cultures during differentiation. After seven days in the differentiation medium, the cells were fixed and immunostained with antibodies against nestin (A-D) and ki-67 (E-H). The proportion of nestin- and ki-67-positive cells showed no significant difference between different groups and was less than 15% (I). The quantification of immunoreactive cells was performed as described in Figure 3-I. *P<0.05. Scale bars: 50 µm.

**Figure 6:**
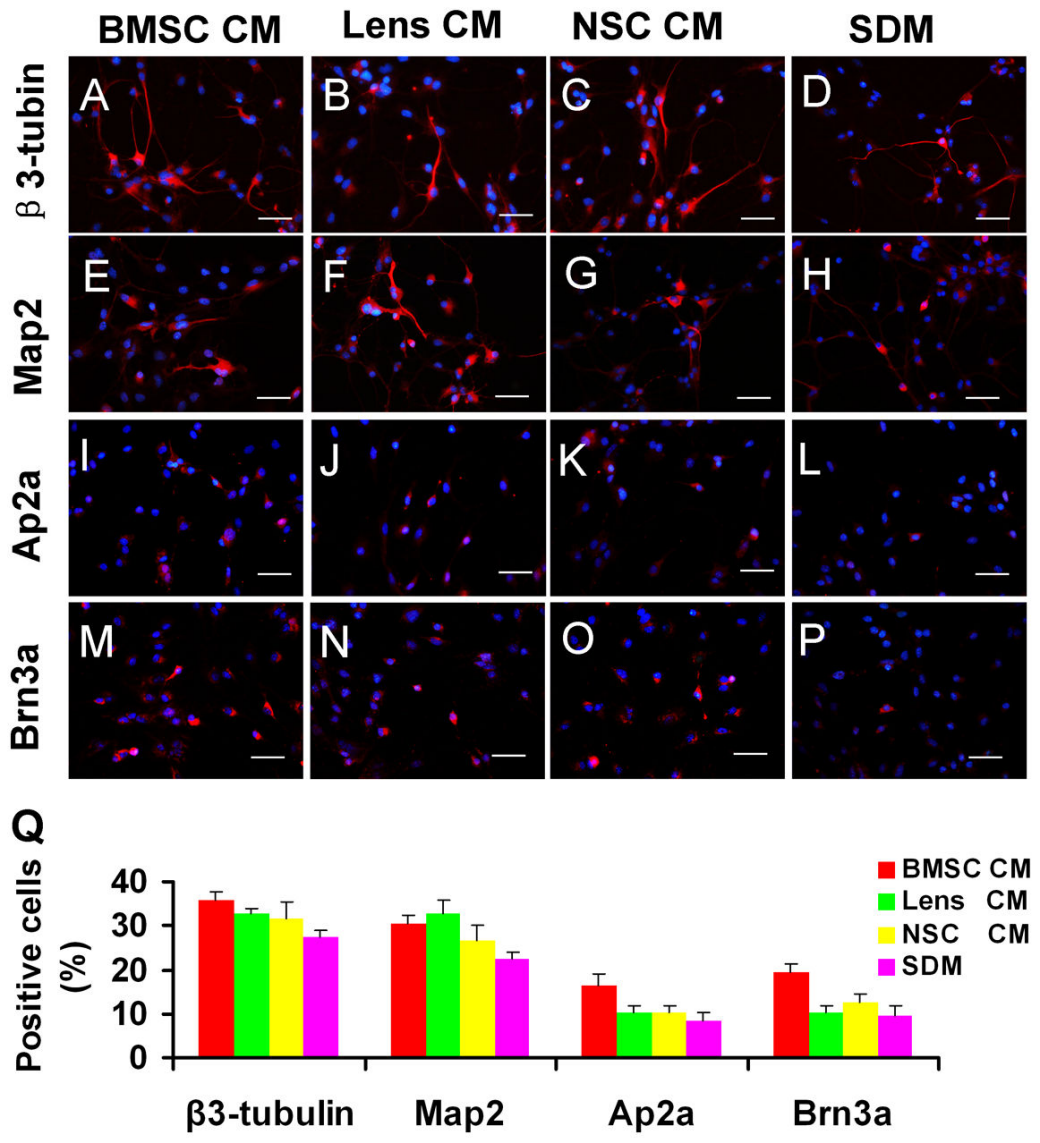
Potential of RPC differentiation towards neurons after exposure to CM. After RPCs were cultured in the differentiation condition for 7 days, the cells were immunolabelled for anti-*β*3-tubulin (A-D), -Map2 (E-H), -AP2α (I-L) and -Brn3a (M-P). The proportion of *β*3-tubulin, AP2α and Brn3a-positive cells was highest in BMSC CM-treated RPCs (Q). The percentage of MAP2-immunoreactive cells was significantly higher in lens and BMSC CM cultures (Q). The quantification of immunoreactive cells was performed as described in Figure 3-I. *P<0.05. Scale bars: 50 µm.

**Figure 7:**
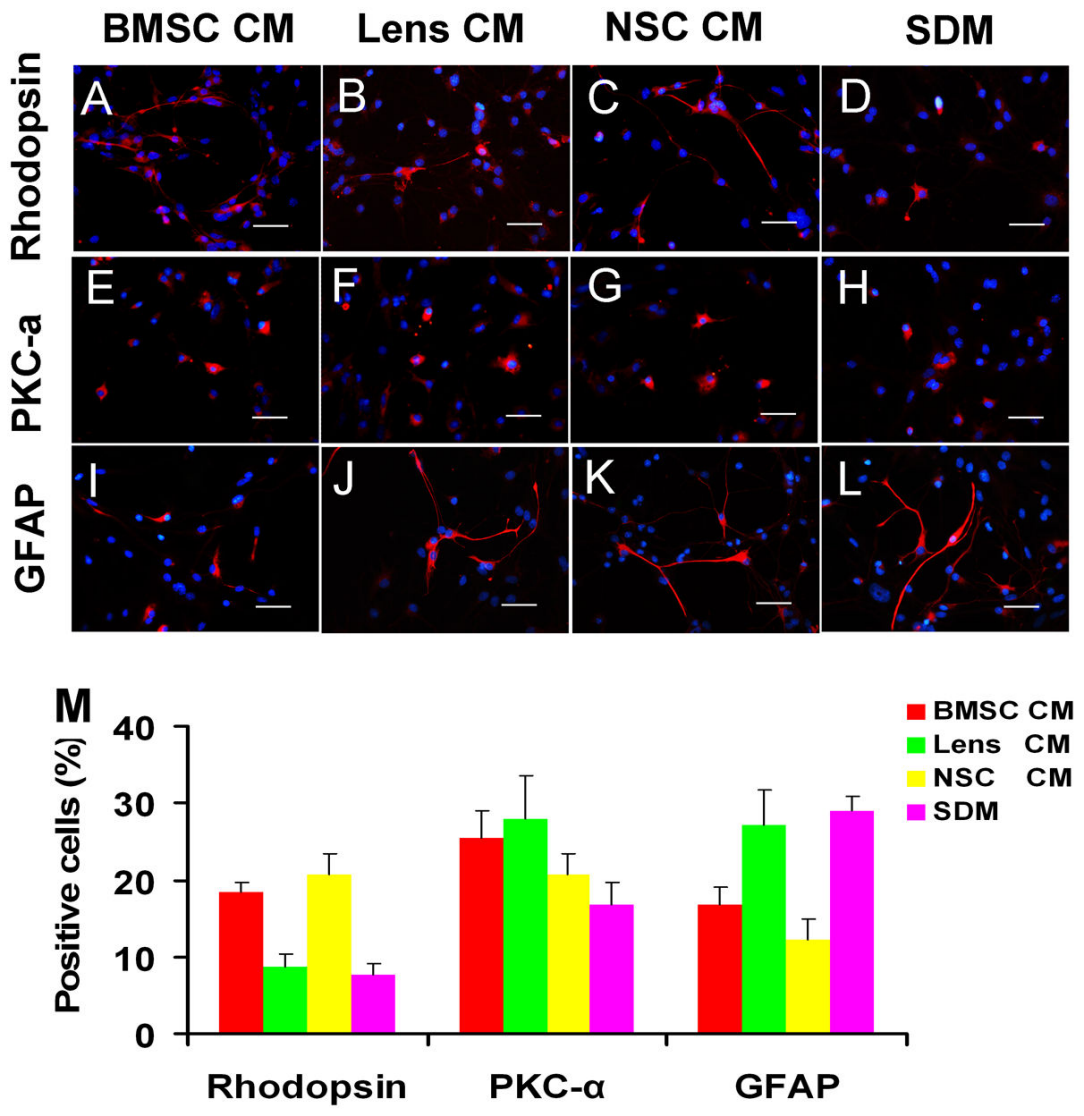
Potential of RPC differentiation towards neuronal and glial cells after exposure to CM. After RPCs were cultured in differentiation medium for seven days, the cells were fixed and immunostained with antibodies against rhodopsin (A-D), PKC-α (E-H) and GFAP (I-L). The percentages of rhodopsin-positive cells were higher in NSC and BMSC CM-treated RPC cultures than in other groups, and PKC-α immunoreactive cells were detected more in CM treated RPCs. However, the ratio of GFAP-positive cells was decreased in the BMSC and NSC CM-treated cultures compared with the controls (M). Quantification of immunoreactive cells was performed as described in Figure 3-I. *P<0.05. Scale bars: 50 µm.

The expression of several essential markers involved in the differentiation of RPCs was also evaluated using immunocytochemistry analysis. The percentages of nestin- and Ki-67-positive cells were markedly decreased (less than 15% for both), with no significant difference between different group cultures under the differentiation conditions (Figure 5A-I) when compared with those in the proliferation conditions (more than 85% and 58% for nestin- and Ki-67-positive cells, respectively) (Figure 3I). This result indicates that the proliferation capacity of RPCs decreased and that most RPCs differentiated into their daughter cells under the differentiation conditions. Our study also showed that the BMSC CM-treated RPC cultures displayed more β3-tubulin-, AP2- and Brn3a-immunoreactive cells than the other groups (36 ± 1.73%, 16.3 ± 2.8% and 19.3 ± 2.1% in BMSC CM; 32.6 ± 1.05%, 10.2 ± 1.5% and 10.4 ± 1.3% in lens CM; 31.7 ± 3. 5%, 10.1 ± 1.6% and 12.6 ± 1.9% in NSC CM; 27.3 ± 1.5%, 8.4 ± 1.8% and 9.5 ± 2.1% in SDM) (Figure 6A-D, I-Q). For MAP-2 (Figure 6E-H, Q) and PKC-α (Figure 7E-H, M), the immunoreactive cell ratios were significantly higher in lens and BMSC CM-treated cultures. The proportions of rhodopsin-positive cells were significantly higher in NSC and BMSC CM-treated cultures than in the control cells (Figure 7A-D, M). In contrast, the ratio of GFAP-labelled cells was clearly decreased in the BMSC CM-treated cultures compared with the control cells (16.7 ± 2.2% and 29.0 ± 1.9%, respectively) (Figure 7I-J, M). These findings are consistent with the qPCR results.

Taken together, these data demonstrated that the bone marrow mesenchymal stem cell-derived conditioned medium (BMSC CM) can enhance the proliferation and neuronal differentiation of RPCs, which may be partly mediated by BMSC CM-derived bFGF and CNTF.

## Discussion

Signals from adjacent differentiated cells and extracellular matrix molecules play an important role in the properties of self-renewal and multilineage differentiation of stem cells [4,19,20]. In the present study, we investigated the effect of BMSC-derived conditioned medium on the expansion and differentiation of RPCs in vitro.

The present study investigated the expression of critical retinal progenitor-related markers, including nestin and Pax6. The transcription factors nestin and Pax6 have been reported to mediate the full retinogenic potential of RPCs and to be required for the maintenance of RPCs in the undifferentiated state [21]. Our present data showed that the expression levels of nestin and Pax6 in the BMSC CM-treated RPC cultures were similar to or greater than those in the controls, indicating that the BMSC CM-treated RPCs retained an undifferentiated state.

In this study, the expression of Ki-67 (a marker for cell proliferation) was significantly higher in the BMSC CM-treated cultures, consistent with our cck-8 analysis data, indicating that RPCs display a dramatic growth promotion when exposed to BMSC-derived conditioned medium during the expansion period. In addition, in the BMSC CM-treated RPC cultures, the expression of TLX and betacellulin (BTC) were clearly upregulated when compared with the controls. Previous studies indicated that TLX and BTC play important roles in promoting neural stem cell proliferation [22,23].

The observed robust growth of the BMSC CM-treated RPCs may have contributions from multiple soluble factors, including BMSC CM-derived mitogenic factor bFGF as shown in present study. Several published reports have indicated that the effect of BMSCs on neurons may be mediated by the supply of cell protective and mitogenic factors in a paracrine manner [24,25]. The other factor likely responsible for this finding may be that the BMSC CM-treated RPCs grow as adherent cells rather than as the spherical clusters in the control cultures. This hypothesis is supported by our previous result that the expansion capacity of RPCs grown in adherent conditions was better than that of cells grown as spherical clusters [26]. Thus, the BMSC CM may stimulate RPC growth through a combination of the supply of cell protective and mitogenic factors and by promoting adherence.

For cell replacement therapy in retinal neurodegenerative diseases, the optimal target is to induce stem/progenitor cells to produce a low proportion of glial cells and a high proportion of retinal neurons. Previous studies have reported that retinal stem/progenitor cells are more inclined to generate glia [4,27]. The present study demonstrated that PRCs exposed to BMSC CM differentiated less often into GFAP-positive glia and were more likely to generate retinal neurons. Our data revealed that the retina-specific interneuronal markers PKC-α and AP2α were highly expressed in BMSC CM-treated RPC cultures. Meanwhile, the retinal ganglion cell (RGC) marker Brn3a and photoreceptor cell marker rhodopsin also showed significantly increased expression levels in the RPC cultures, indicating that the BMSCs can stimulate RPC differentiation towards retinal interneurons, photoreceptors and ganglion cells. These observations are consistent with previous studies showing that BMSCs provide instructive signals that can direct the neuronal differentiation of neural stem cells (NSCs) and promote axonal growth when BMSCs and NSCs are co-cultured in vitro [11,28]. In addition, our present study showed that CNTF was enriched about 3 fold in conditioned media from BMSCs compared to RPCs. An upregulation in the expression of MAP-2, AP2α, Brn3a and rhodopsin was detected in the CNTF-treated RPC cultures under differentiation conditions, which was supported with a previous report [4], indicating that the effect of BMSC CM on RPC differentiation may be partly mediated by BMSC CM-derived CNTF.

In summary, our findings demonstrate that BMSCs supported RPC neuronal differentiation, indicating a means for optimizing RPC cell fate determination and allowing potential efficient production of specific retinal neurons for use in future neuroretinal cell replacement therapies.

## Conclusion

The present study investigated the effect of BMSC CM on the proliferation and multipotentiality of RPCs. Our data demonstrated that BMSC CM can enhance RPC proliferation and keep RPCs in an undifferentiated state. More interestingly, treatment with BMSC CM favoured RPC differentiation towards retinal neurons, including photoreceptors and RGCs. These findings suggest that the BMSC CM can profoundly influence the proliferation and differentiation of RPCs, which may be partly mediated by BMSC CM-derived bFGF and CNTF. However, further investigation is needed to determine how BMSCs promote RPC proliferation and stimulate RPC neuronal differentiation.

## Supporting Information

Figure S1 **ELISA analysis of BMSC CM-derived bFGF and CNTF.** The conditioned medium from BMSCs was substantially enriched for bFGF and CNTF compared to the RPCs-cultured medium.
(TIF)

Figure S2 **The effect of bFGF or CNTF on RPC proliferation capacity.** Under proliferation conditions, the expansion capacity of the cells was evaluated by CCK-8 analysis. In comparison with the cells treated with BMSC CM, the expansion capacity of the RPCs cultured in the presence of BMSC CM plus anti-bFGF was partly inhibited. Addition of bFGF to the RPC cultures in SM, RPCs exhibited obvious increase in expansion capacity as compared with the cells in SM (without bFGF) (A). In addition, the obvious effect of CNTF or anti-CNTF on RPC expansion capacity was not detected (B).
(TIF)

Figure S3 **Effect of CNTF on RPC differentiation.** After RPCs were cultured in the differentiation conditions for 7 days, qPCR analysis showed that the RPCs treated with BMSC CM plus anti-CNTF displayed slightly low expression of MAP-2, AP2α, Brn3a and rhodopsin compared with the cells in BMSC CM, while these retinal neuronal marker expression levels were upregulated in the RPCs treated with SDM plus CNTF compared with the cells in SDM only.
(TIF)
