# Supplementary figures and images for: Bone Marrow Mesenchymal Stem Cells Stimulate Proliferation and Neuronal Differentiation of Retinal Progenitor Cells
Source: PLoS One. 2013 Sep 30;8(9):e76157. doi: 10.1371/journal.pone.0076157 (PMC3786983; doi:10.1371/journal.pone.0076157)

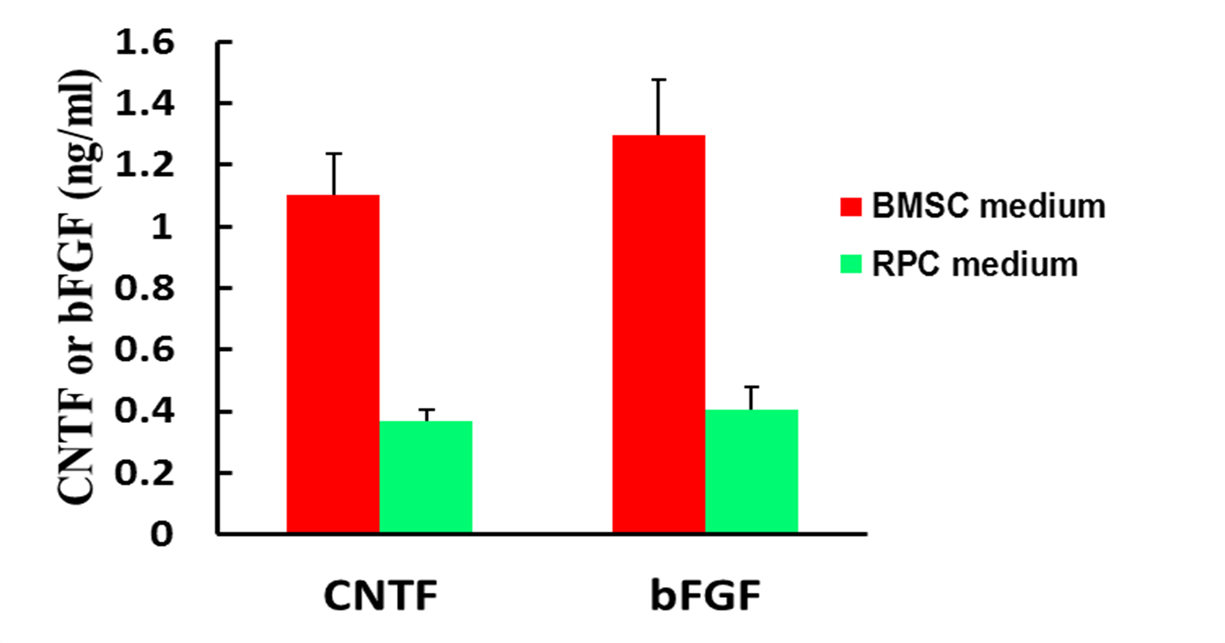

Supplement: Figure S1 — ELISA analysis of BMSC CM-derived bFGF and CNTF. The conditioned medium from BMSCs was substantially enriched for bFGF and CNTF compared to the RPCs-cultured medium. (TIF) [file pone.0076157.s001.tif]

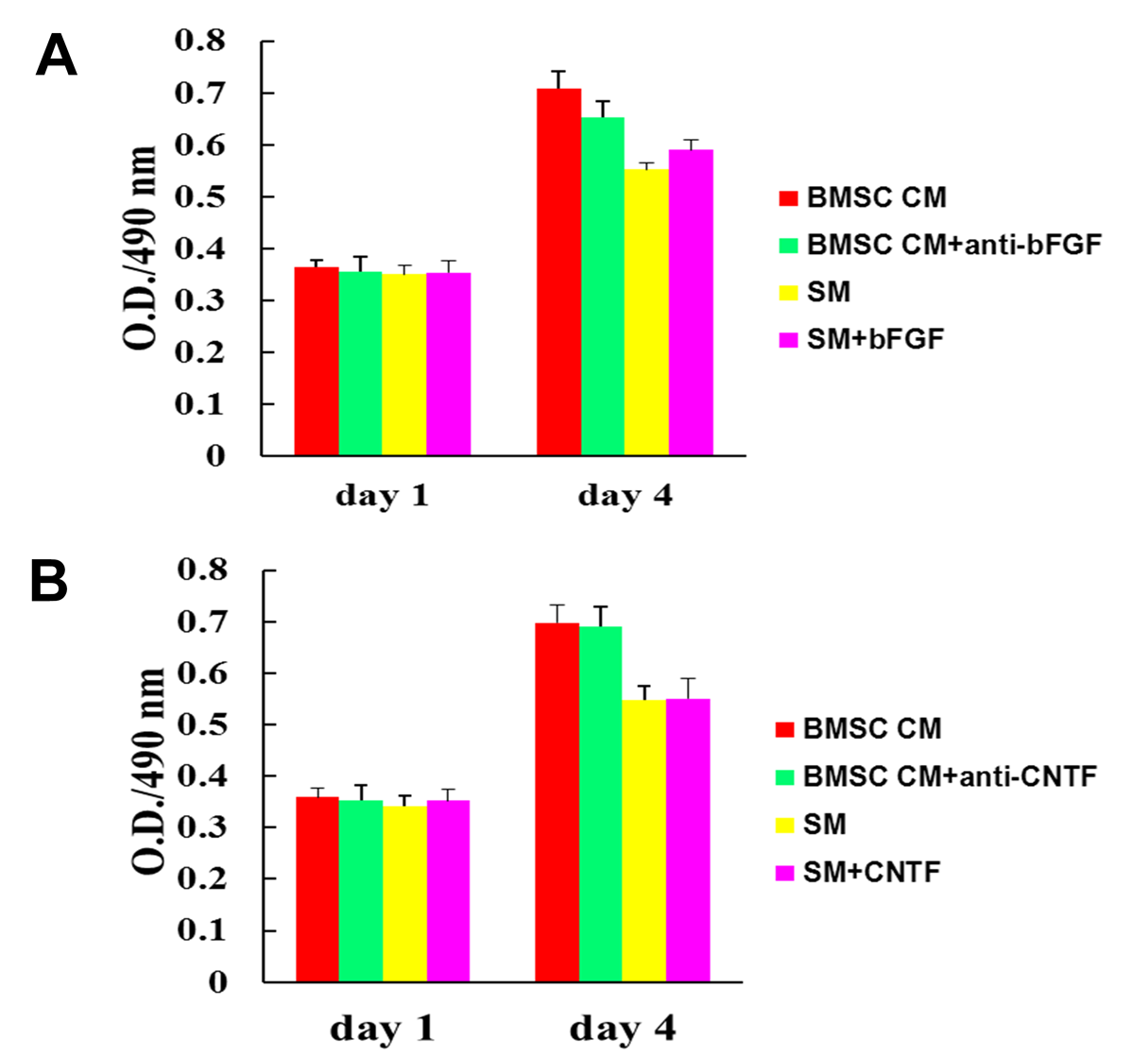

Supplement: Figure S2 — The effect of bFGF or CNTF on RPC proliferation capacity. Under proliferation conditions, the expansion capacity of the cells was evaluated by CCK-8 analysis. In comparison with the cells treated with BMSC CM, the expansion capacity of the RPCs cultured in the presence of BMSC CM plus anti-bFGF was partly inhibited. Addition of bFGF to the RPC cultures in SM, RPCs exhibited obvious increase in expansion capacity as compared with the cells in SM (without bFGF) (A). In addition, the obvious effect of CNTF or anti-CNTF on RPC expansion capacity was not detected (B). (TIF) [file pone.0076157.s002.tif]

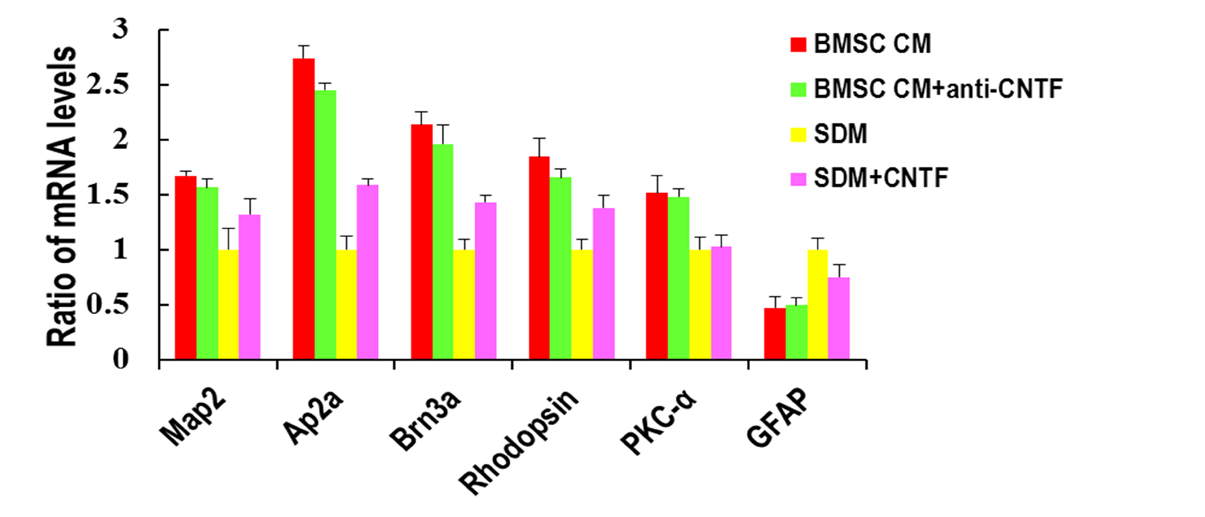

Supplement: Figure S3 — Effect of CNTF on RPC differentiation. After RPCs were cultured in the differentiation conditions for 7 days, qPCR analysis showed that the RPCs treated with BMSC CM plus anti-CNTF displayed slightly low expression of MAP-2, AP2α, Brn3a and rhodopsin compared with the cells in BMSC CM, while these retinal neuronal marker expression levels were upregulated in the RPCs treated with SDM plus CNTF compared with the cells in SDM only. (TIF) [file pone.0076157.s003.tif]
